# Genome Insights into the Novel Species *Microvirga brassicacearum*, a Rapeseed Endophyte with Biotechnological Potential

**DOI:** 10.3390/microorganisms7090354

**Published:** 2019-09-14

**Authors:** Alejandro Jiménez-Gómez, Zaki Saati-Santamaría, José M. Igual, Raúl Rivas, Pedro F. Mateos, Paula García-Fraile

**Affiliations:** 1 Microbiology and Genetics Department, University of Salamanca, 37007 Salamanca, Spain; alexjg@usal.es (A.J.-G.); zakisaati@usal.es (Z.S.-S.); raulrg@usal.es (R.R.); pfmg@usal.es (P.F.M.); 2 Spanish-Portuguese Institute for Agricultural Research (CIALE), Villamayor, 37185 Salamanca, Spain; 3 Associated R&D Unit, USAL-CSIC (IRNASA), 37007 Salamanca, Spain; mariano.igual@csic.es; 4 Institute of Natural Resourses and Agrobiology of Salamanca (IRNASA-CSIC), Salamanca 37008, Spain

**Keywords:** plant–bacteria interactions, genome mining, PGPB, biofertilizer, CAZymes, secondary metabolites, hydrolytic enzymes

## Abstract

Plants harbor a diversity of microorganisms constituting the plant microbiome. Many bioinoculants for agricultural crops have been isolated from plants. Nevertheless, plants are an underexplored niche for the isolation of microorganisms with other biotechnological applications. As a part of a collection of canola endophytes, we isolated strain CDVBN77^T^. Its genome sequence shows not only plant growth-promoting (PGP) mechanisms, but also genetic machinery to produce secondary metabolites, with potential applications in the pharmaceutical industry, and to synthesize hydrolytic enzymes, with potential applications in biomass degradation industries. Phylogenetic analysis of the 16S rRNA gene of strain CDVBN77^T^ shows that it belongs to the genus *Microvirga*, its closest related species being *M. aerophila* DSM 21344^T^ (97.64% similarity) and *M. flavescens* c27j1^T^ (97.50% similarity). It contains ubiquinone 10 as the predominant quinone, C19:0 cycloω8c and summed feature 8 as the major fatty acids, and phosphatidylcholine and phosphatidylethanolamine as the most abundant polar lipids. Its genomic DNA G+C content is 62.3 (mol %). Based on phylogenetic, chemotaxonomic, and phenotypic analyses, we suggest the classification of strain CDVBN77^T^ within a new species of the genus *Microvirga* and propose the name *Microvirga brassicacearum* sp. nov. (type strain CDVBN77^T^ = CECT 9905^T^ = LMG 31419^T^).

## 1. Introduction

After millions of years of evolution, microorganisms have compiled a noteworthy genetic diversity with biotechnological potential, which can be additionally enhanced by tools, such as genetic engineering or heterologous gene expression. Thus, microbial isolates are essential for diverse biotechnology-based industries, such as pharmaceutical biotechnology (where they play important roles in the production of antibiotics, antivirals, anticarcinogenic compounds, etc.), agricultural biotechnology (where they are applied as biocontrol agents or probiotics for plant growth promotion), and environmental biotechnology (with variate applications such as lignocellulose breakdown or degradation of xenobiotic compounds). Moreover, according to recent estimations, potentially 99.999% of microbial taxa remain undiscovered [1], so there is a huge genetic potential yet undiscovered.

In the last couple of decades, the extensive use of the PCR and sequencing of the 16S rRNA gene has allowed a more accurate bacterial identification and served as the base for the discovery of new taxa, being the gold standard for prokaryote identification. According to Jongsik and collaborators [2], a bacterial strain with a similarity under 98.7% in the 16S rRNA gene sequence with the type strain of its closest related species represents a novel species and, in case of a similarity over 98.7%, a value under 70% in total DNA–DNA hybridization (DDH) also indicates a novel species [3]. More recently, the quick development of DNA sequencing platforms that generate rapid and low-cost high-throughput sequencing data has spearheaded the widespread sequencing of bacterial genomes which, together with the development of bioinformatics tools for genome sequence comparisons, facilitates the process of new species identification, by means of genome-to-genome sequence comparison [2].

Genome sequences also allow us to mine the genetic potential of a microorganism for different purposes, for example, plant growth promotion mechanisms [4] and the production of secondary metabolites with applications in the pharmaceutical industry [5] or carbohydrate-active enzymes (CAZymes) with several biotechnologically based industry uses, such as biomass degradation, biodiesel production, or bioremediation [6].

Plants are the ecological niche of a broad range of microorganisms which constitute their microbiome [7]. Some of these microorganisms establish symbiotic associations with the plant, supplying their host with nutrients or improving its fitness by the production of phytohormones or the induction of resistance to abiotic stresses [8]. These plant growth-promoting (PGP) microorganisms can be applied as biofertilizers or plant probiotics to improve crop yields in an ecologically friendly manner and thus, they are produced and formulated as bioinoculants in the industry. Nevertheless, plants are an underexplored niche as a source of microorganisms for other biotechnological applications different from those of the agro-based industry.

The genus *Microvirga* is a cosmopolitan genus which as of now includes 17 validated species isolated from a wide diversity of niches, such as human stool [9], air [10], thermal water [11], natural, domestic, and contaminated soils [12,13,14,15,16,17], and even as legume nodule endophytes [18,19,20]. Some studies report the genetic potential of some strains classified within the *Microvirga* genus for arsenic oxidation [15] and the production of pigments and amylolytic enzymes [21].

In this study, we propose the description of *Microvirga brassicacearum* sp. nov. isolated as a rapeseed bacterial endophyte with the capability to promote plant growth. Moreover, we obtained and mined the genome sequence of strain *M. brassicacearum* CDVBN77^T^, showing its genetic potential not only as a plant growth promoter but also as a producer of secondary metabolites with a potential interest in the pharmaceutical industry as well as of enzymatic machinery for biomass degradation.

## 2. Materials and Methods

### 2.1. Strain Isolation and Identification

Strain CDVBN77^T^ was isolated as part of a collection of rapeseed bacterial root endophytes from the roots of rapeseed plants harvested in the locality of Castellanos de Villiquera (province of Salamanca, Spain) [22]. Plants in the phenological state of rosette were collected and kept refrigerated to be transported to the lab. Root endophytes were obtained after washing with sterile water. The plants were extracted from the soils, kept refrigerated, and shipped to the laboratory. To isolate rapeseed root bacterial endophytes, roots were washed in sterile Petri dishes containing sterile water (10 times) and then sterilized by immersion in sodium hypochlorite (2%) for 2 min. Afterward, surfaced sterilized roots were washed in sterile water (5 times) and dried without using sterile filter paper.

Surface sterilized roots were smashed with a pestle in a sterile mortar and the extract was serially diluted in sterile water. Then, 100 µL of the 10^−2^, 10^−3^, and 10^−4^ dilutions were plated onto Petri dishes containing different media: YMA (Microkit®, Laboratorios Microkit, Madrid, Spain), Tryptic Soy Agar (TSA) (Difco^TM^, Difco Laboratories, Le Pont de Claix, France), 869 medium (Tryptone (10 g/L), yeast extract (5 g/L), NaCl (5 g/L), D-glucose (1 g/L), CaCl_2_ (0.345 g/L), and agar (20 g/L)), and ten times diluted 869 medium (869(1/10) medium). Plates were incubated at 28 °C for 21 days. The emerging bacterial colonies were regularly isolated to obtain pure cultures. Then, for long-term storage, isolated strains were stored in a sterile 20% glycerol solution at −80 °C.

### 2.2. DNA Extraction and 16S rRNA Amplification and Sequencing

CDVBN77^T^ DNA for amplifying and sequencing 16S rRNA gen was extracted using the REDExtract-n-AmpTM Plant PCR Kit (Sigma-Aldrich, Darmstadt, Germany) following the manufacturer’s instructions. The amplification was performed using 2 µL of bacterial DNA (100 µg/mL) as the template, 6 µL of Milli-Q sterile water, 12.5 µL REDExtract Ready Mix, 2.5 µL 27F 1 µM (5’ GAGGGTGGCGGTTCT 3’), and 2,5 µL 1522R 1 µM (5’AAGGAGGTGATCCANCCRCA 3’). The PCR conditions were the following: a first step of pre-heating at 95 °C for 9 min, 35 cycles of denaturing at 94 °C for 1 min; annealing at 56.5 °C for 1 min 30 s and extension 72 °C for 2 min, and a final extension at 72 °C for 7 min.

PCR product was purified using DNA Cleanup Micro Kit (Thermo Scientific^TM^, Göteborg, Sweden). The sequence reaction was performed on an ABI PRISM sequencer (Applied Biosystem^TM^, Foster, CA, USA) using a Dye Terminator Cycle Sequencing Ready Reaction Kit (University of Salamanca, Salamanca, Spain). The isolate strain was identified using the following databases: NCBI’s BLASTn program [23] (https://blast.ncbi.nlm.nih.gov/Blast.cgi?PAGE_TYPE=BlastSearch) and EzTaxon tool [24].

### 2.3. Draft Genome Sequencing and Annotation

Bacterial DNA for genome sequencing was obtained from cells grown in YMA medium at 28 °C for five days. DNA extraction was performed with the Quick DNA^TM^ Fungal/Bacterial Miniprep Kit (Zymo Research®, Irvine, CA, USA) following the protocol provided by the manufacturer.

The draft genome sequence was obtained on an Illumina platform from the Genomics Service of the Institute of Functional Biology and Genomics (IBFG) (Salamanca, Spain). Sequences were assembled using Velvet 1.12.10, [25]. Gene calling, annotation, and search of genes related to plant growth promotion capabilities were performed using RAST Pipeline [26]; the BlastKOALA tool was also used to perform KEGG Orthology (KO) assignment based on the KEGG database [27] in order to complete the mining of genes related to plant growth promotion; the dbCAN2 meta server [28] was used to identify the coding sequences (CDSs) encoding carbohydrate-active enzymes (CAZymes) using HMMER annotation; and the analysis of gene clusters related to secondary metabolites production was performed using antiSMASH 5.0 webserver [29]. This genome sequence was deposited at the DDBJ/EMBL/GenBank under the accession VCMV00000000. The version described in this paper is version VCMV01000000.

### 2.4. Genotypic Analysis of Microvirga brassicacearum CDVBN77^T^

The GenBank accession number of the CDVBN77^T^ 16S rRNA gene sequence is MK938302. Phylogenetic analysis of the CDVBN77^T^ 16S rRNA gene sequence and the sequences of the type strains of all the species included within the genus *Microvirga* was performed using MEGA7 software [30], based on the alignment of the sequences with Clustal_W [31]. The distances were calculated using Kimura’s two-parameter model [32], and the phylogenetic tree was inferred using neighbor-joining (NJ; [33]) analysis. The average nucleotide identity (ANI) and digital DNA–DNA hybridization (dDDH) values between the draft genome sequence of strain CDVBN77^T^ and those of its closest related species *M. aerophila* c27j1^T^ (GenBank/EMBL/DDBJ accession number NZ_QOIO00000000.1) and *M. flavescens* DSM 21344^T^ (GenBank/EMBL/DDBJ accession number QMKH00000000) were calculated using the ANI calculator (www.ezbiocloud.net/tools/ani) and the Genome-to-Genome Distance Calculator (GGDC 2.1) (http://ggdc.dsmz.de/distcalc2.php), respectively. The mol % G+C content of DNA was determined from the draft genome sequence. 

### 2.5. Chemotaxonomic Analysis of Microvirga brassicacearum CDVBN77^T^

For the analysis of fatty acid methyl esters (FAMEs), biomass was harvested as detailed by Weon et al. [8]. For the analyses of polar lipids and respiratory quinones, biomass was harvested after cultivation of the bacterium for seven days in R2A broth medium (dextrose (0.5 g/L), dipotassium phosphate (0.3 g/L), casamino acids (0.5 g/L), magnesium sulphate (0.05 g/L), proteose peptone (0.5 g/L), sodium pyruvate (0.3 g/L), starch (0.5 g/L), and yeast extract (0.5 g/L)) at 28 °C and 150 rpm shaking. Analysis of FAMEs was performed as previously described [34]. For the identification of polar lipids and respiratory quinones, cells collected from the plates into sterile Falcon® tubes were freeze dried and sent to the Identification Service of the German Collection of Microorganisms and Cell Cultures DSMZ.

### 2.6. Microvirga brassicacearum CDVBN77^T^ Bacterial Characterization

Catalase and oxidase tests [35] and Gram staining [36] were performed using previously described protocols.

Cell morphology and flagella were observed under a Zeiss EM209 transmission electron microscope, after growth in YMA medium at 28 °C for five days and cell treatment with 2% uranyl. To assay salt tolerance, the bacterium was cultivated in YMB medium (mannitol (7.0 g/l), yeast extract (2 g/l), dipotassium phosphate (0.2g/l), and magnesium sulphate (0.2g/l)) supplemented with 0, 0.5, 1, 1.5, and 2% (w/v) NaCl. The same medium adjusted to a final pH range of 3–11 was used to examine the growth capability at different pHs; in both cases, flasks containing 50 mL of the different media were inoculated with a 10 µl sterile plastic loop containing cells collected from a two-day plate culture of the bacterium. After the inoculation, flasks were cultivated in a shaker at 150 rpm for two days at 28 °C. Plates containing YMA medium (YMB plus 2% agar) were incubated at 4, 12, 25 and 37 °C to determine the temperature range for optimal growth. In all cases, the cultures were examined at 2, 5, 10, and 15 days after inoculation.

Enzymatic assays were performed using the *p*-nitrophenyl (pNP) substrates listed in Table 1. Substrates were used at a concentration of 0.2% in 50 mM phosphate buffer (pH 7); however, those requiring pH 8.5 were prepared in Tris-HCl 0.2 M buffer and those requiring pH 5.0 were prepared in an acetate buffer (0.2 M). The enzymatic reactions were carried out in a multi-well plate mixing 50 μL of substrate prepared as specified above and 50 μL of a suspension of bacteria in sterile water with 6 × 10^9^ CFU/mL obtained from cells previously grown in YMA medium for 15 days at 28 °C. Plates with the reaction mixture were incubated at 28 °C for 24 h and then revealed with 100 μL per well of a 4% sodium carbonate solution. A positive reaction was evidenced by the appearance of a yellow color within a few seconds.

To evaluate siderophore production, strain CDVBN77^T^ was inoculated in M9-CAS-agar medium plates [37] with the modifications suggested by Alexander and Zuberer [38] after 21 days of growth at 28 °C. The addition of hexadecyltrimethylammonium bromide (HDTMA) as a cationic solvent was used to stabilize the Fe-CAS complex, which provides the characteristic orange-colored halo around the colonies when siderophores are produced.

The solubilization of non-soluble phosphates into soluble assimilable ions was analyzed in Pikovskaya medium plates [39], which contain calcium phosphate (Ca_2_HPO_4_) as P source. The presence of clear halos surrounding bacterial colonies are indicative of phosphate solubilization. Strain CDVBN77^T^ was inoculated in Pikovskaya medium and phosphate solubilization was evaluated after 21 days of growth at 28 °C.

Cellulose production was determined as described by Robledo et al. [40] in Nutrient Agar (NA) plates containing Congo Red colorant, which binds to cellulose polymers. Red-colored colonies are indicative of cellulose production. Strain CDVBN77^T^ was inoculated in plates with Congo Red and the color of the colonies was evaluated after 21 days of growth at 28 °C.

Other phenotypic characters were tested by using the API 20NE and API ZYM (bioMerieux^®^) miniaturized galleries following the manufacturer’s instructions.

## 3. Results

### 3.1. Bacterial Isolation and Identification

Strain CDVBN77^T^ was isolated as part of a collection of rapeseed root endophytes from plants sampled at the municipality of Castellanos de Villiquera (province of Salamanca, Spain) [22]. The comparison of the 16S rRNA gene sequence with those of type strains of described species available in GenBank and EzBiocloud databases showed that all strains but CDVBN77^T^ showed similarities higher than 98% with their closest related type strains, whereas the similarity of strain CDVBN77^T^ with *M. aerophila* DSM 21344^T^, its closest related type strain, was only 97.64%, which according to the threshold value of 98.7% in the 16S rRNA gene sequence with the closest related species indicated by Jongsik and collaborators [2] indicates that the strain constitutes a new species within the genus *Microvirga*.

Therefore, we performed a phylogenetic analysis of the 16S rRNA gene sequence of strain CDVBN77^T^ and those of the type strains of all species included within the genus *Microvirga*.

**Figure 1:**
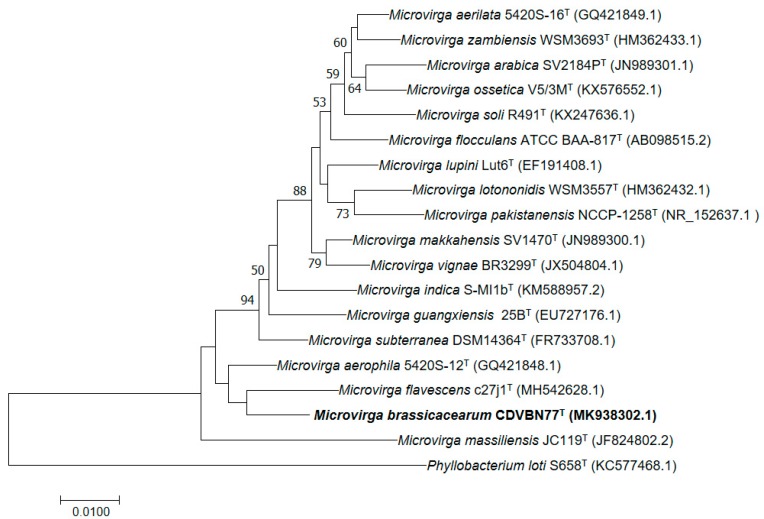
Neighbor-joining phylogenetic tree based on the nearly complete 16S rRNA gene sequences of all species classified within the genus *Microvirga* and the species *Phylobacterium loti*, which was included as an outgroup. The numbers are bootstrap values indicating the significance of the branches calculated as a percentage for 1000 subsets. Bar: 1 nt substitutions per 100 nt. Accession numbers of the sequences are indicated in parentheses.

Phylogenetic trees constructed with both Maximum Likelihood (ML) and Neighbor Joining (NJ) methods showed a similar topology (Figure 1) with strain CDVBN77^T^ appearing in a separated branch with *M. flavescens* c27j1^T^ and *M. aerophila* DSM 21344^T^ as the closest related species. Thus, the phylogenetic analysis of the 16S rRNA gene supports the classification of the bacterium CDVBN77^T^ as a new species within the genus *Microvirga.*

### 3.2. Genome Properties and Comparison with Those of Its Closest Related Species

The genome sequence of strain CDVBN77^T^ was obtained in order to be compared with those of its closest related species. Genome properties of the strain based on RAST annotations are detailed in Table 2.

ANI values between the genome sequence of CDVBN77^T^ and *M. flavenscens* c27j1^T^ and *M. aerophila* DSM 21344^T^ were 76.14% and 77.23%, respectively. Considering the recommended ANI threshold value for species delimitation of 95% to 96% [41], these genome comparisons clearly support the classification of strain CDVBN77^T^ as a novel species of the genus *Microvirga*.

The results of dDDH between the genome sequence of CDVBN77^T^ and *M. flavescens* c27j1^T^ and *M. aerophila* DSM 21344^T^ were 20.50% and 21.20%, respectively. Since the dDDH threshold value for species delineation has been established at 70% [42], we can conclude that the bacterial isolate CDVBN77^T^ does not belong to its closest related species and should be classified within a new species of the genus *Microvirga*.

### 3.3. Plant Growth Promotion Potential

The genome mining revealed many genes involved in PGP mechanisms. Regarding the acquisition of nutrients, we found some mechanisms involved in the solubilization of P and K, making their uptake easier for the plant. The genome annotations showed genes encoding for proteins with phosphatase activity, one of the main mechanisms for P solubilization, such as the enzymes alkaline phosphatase (EC 3.1.3.1), exopolyphosphatase (EC 3.6.1.11), inorganic triphosphatase (EC 3.6.1.25) and pyrophosphatase (EC 3.6.1.1), polyphosphate kinase (EC 2.7.4.1), and a pyrophosphate-energized proton pump (EC 3.6.1.1) [43]. The glucose dehydrogenase PQQ-dependent enzyme (EC 1.1.5.2), which is responsible of the oxidation of glucose into the phosphate solubilizing molecule gluconic acid [44,45], was also found in this search. In addition, some other enzymes involved in the production of P and K solubilization acids are encoded by this bacterium, such as citrate synthase (EC 2.3.3.1) and malate synthase G (EC 2.3.3.9) [46]. Moreover, regarding the transport of these two elements, the genome encodes transport systems such as the Pst system for P [47] or the Kup and Kef systems for K [48]. Regarding the iron supply, we found some genes annotated as siderophores or other iron-associated transport proteins, such as the ferrichrome-iron receptor, a ferric iron ABC transporter, a ferric hydroxamate ABC transporter (TC 3.A.1.14.3), the ATP-binding protein FhuC, as well as proteins of the Fec system, the AfuABC system (Iron (III)), and the SitA system (Iron(II)/manganese). Finally, the capability to fix atmospheric nitrogen into a plant’s available state of nitrogen [49] is a common mechanism in rhizobial strains. In this case, we found the presence of genes implicated on Fix and Nif systems.

A prior step to endophytic living is to attach and form biofilms over the root surface. Bacteria commonly produce exopolysaccharides in order to colonize the root surface [50]. Genome annotations revealed a great number of genes related to the synthesis or transport of exopolysaccharides, for example, the *exoD* gene, which is associated with this process [51], *lpx* genes, responsible for the synthesis of this type of molecules [52], or lpt [53] and Rf [54] transport systems. Once inside the plant, the bacterium must evade the plant defense. The genome annotations revealed that the bacterium encodes genes for the production of salicylate 1-hydroxylase (EC 1.14.13.1), an enzyme that degrades the salicylic acid into catechol.

Moreover, strain CDVBN77^T^ grew on chrome azurol S (CAS) indicator medium, where the colonies were surrounded by a yellow-orange halo (2 mm radius around colonies), indicating siderophore production.

In addition, CDVBN77^T^ solubilized phosphate on Pikovskaya’s agar, forming halos around its colonies (2 mm radius around colonies). Additionally, the results obtained using pNP-based substrates showed the production of phosphatases (with activity at acid, alkaline, and neutral pH) and bisphosphatases (with activity at alkaline pH).

Furthermore, strain CDVBN77^T^ colonies were red after seven days of incubation on plates containing Congo Red. The intensity of the color was comparable to that recorded for other strains, which indicated that CDVBN77^T^ produces a moderate amount of a polysaccharide with 1,4-β-glycosidic bonds.

### 3.4. Potential for the Production of Enzymes with Biotechnological Potential

Analysis of the genome sequence of strain *M. brassicacearum* CDVBN77^T^ with dbCAN2 showed 115 genes encoding different CAZymes involved in degradation, modification, or creation of glycosidic bonds.

In summary, we identified gene modules belonging to five different enzyme classes: (i) glycoside hydrolases (GHs), enzymes that catalyze the hydrolysis of glycosidic linkage of glucoside—30 gene counts in 15 different families; (ii) glycosyltransferases (GTs), involved in the formation of glycosidic bonds—46 gene counts in 11 different families; (iii) polysaccharide lyases (PLs), which perform non-hydrolytic cleavage of glycosidic bonds—2 gene counts in 2 different families; (iv) carbohydrate esterases (CEs), which hydrolyze carbohydrate esters—22 gene counts in 6 different families; and (v) auxiliary activities (AAs), redox enzymes that act in conjunction with CAZymes—15 gene counts in 5 different families (Table 3).

The main enzymes acting in the lignocellulosic breakdown are GH. GHs in the genome sequence of *M. brassicacearum* CDVBN77^T^ belong to the families 1, 2, 3, 15, 16, 23, 25, 31, 63, 102, 103, 105, 108, 109, and 113. Regarding enzymes related to biomass hydrolysis, we found five genes belonging GH family 1, which includes among other enzymes β-glucosidases (EC 3.2.1.21), exoglucanases (EC 3.2.1.74), and 1,4-β-xylosidases (EC 3.2.1.37), two genes belonging to GH family 2, which includes α-L-arabinofuranosidases (EC 3.2.1.55) and 1,4- β-xylosidases (EC 3.2.1.37), and two genes belonging to GH family 3, which includes β-glucosidases (EC 3.2.1.21), exoglucanases (EC 3.2.1.74), 1,4-β-xylosidases (EC 3.2.1.37), and α-L-arabinofuranosidases (EC 3.2.1.55). The enzymes β-glucosidases (EC 3.2.1.21) and exoglucanases (EC 3.2.1.74) belong to the cellulase complex, and 1,4-β-xylosidases (EC 3.2.1.37) and α-L-arabinofuranosidases (EC 3.2.1.55) are implicated in hemicelluloses hydrolysis, cellulose and hemicelluloses being the most abundant plant polymers. β-glucosidases (EC 3.2.1.21) and 1,4-β-xylosidases (EC 3.2.1.37) appear annotated in RAST and BlastKOALA annotations of the CDVBN77^T^ genome sequence. The presence of genes encoding these enzymes suggests that this bacterium could have biotechnological potential for the production of enzymes for lignocellulosic biomass breakdown. 

Moreover, enzymatic assays based on pNP-based substrates show the capability of the strain to synthesize β-galactosidase, β-glucosidase, and α-rhamnosidase. Nevertheless, in the conditions assayed, we could not detect the production of xylosidases or arabinofuranosidases.

### 3.5. Genome Mining of Gene Clusters Associated to the Biosynthesis of Secondary Metabolites

AntiSMASH output revealed five biosynthetic gene clusters (BGCs) involved in the secondary metabolism of the bacterium.

One of those clusters encodes an undescribed terpene BGC in which the core gene is a phytoene synthase (EC 2.5.1.32), an enzyme involved in the first steps of the biosynthesis of carotenoids. According to antiSMASH clusterblast, this cluster seems to be distributed among different *Ochrobactrum* and *Brucella* genomes, but it does not appear in the seven genomes of strains belonging to the *Microvirga* genus included in the database at the moment of writing this work.

Another BGC found in *Microvirga brassicacearum* CDVBN77^T^ is related to the synthesis of a N-acyl-homoserine lactone. This family of molecules is often involved in bacterial quorum sensing [55]. In this case, the cluster is present in other *Microvirga* genomes, but it has never been tested for the production of any specific molecule.

*Microvirga brassicacearum* CDVBN77^T^ also encodes two nonribosomal peptide synthetases (NRPSs) BGCs that are neither described for the production of an already known molecule. Moreover, none of them share similitude with any region of any other *Microvirga* strains’ genomes, although it appears related to sequences of strains belonging to *Pseudomonas* and *Chelatococcus* genera (with 6%–20% genes showing similarity).

In addition, a type III polyketide synthase (T3PKS) is present, a type of BGC involved in the synthesis of a great diversity of molecules derived from the metabolism of fatty acids [56]. The region encoding this BGC shares certain similitude with regions of some *Pseudomonas* genomes but not in the core genes, and none of them has been described for the production of a known metabolite.

### 3.6. Colony and Cellular Morphology in Strain CDVBN77^T^

**Figure 2:**
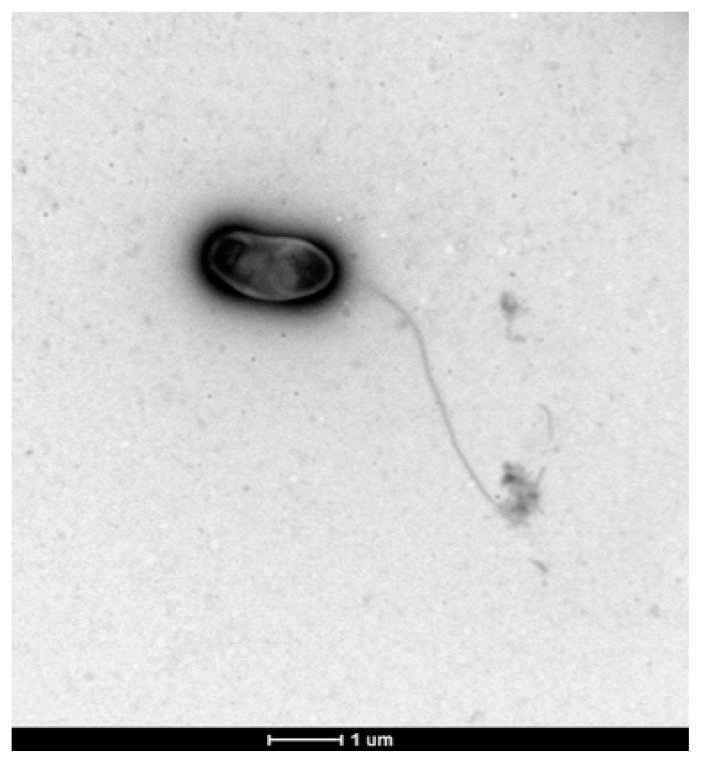
Electron TEM micrograph showing bacterial shape and size and the flagellum of *Microvirga brassicacearum* CDVBN77^T^.

After seven days of incubation at 28 °C in R2A agar medium, strain CDVBN77^T^ forms circular, flat, and transparent colonies with entire borders. The cells are rod-shaped, 1.2–1.5 µm in length and 0.7–0.9 µm in width, and motile by means of a polar flagellum (Figure 2).

### 3.7. Phenotypic and Chemotaxonomic Characterization of Strain CDVBN77^T^

The analysis of respiratory quinones revealed that ubiquinone-10 (Q-10) is the major quinone in CDVBN77^T^, and the main polar lipids of this strain are supporting the classification of the bacterium within the genus *Microvirga* [11].

The major fatty acids in strain CDVBN77^T^ are C_19:0_ cyclo ω8c (24.3%) and Summed Feature 8 (39.3%) (Table 4), as it occurs for its closest related strains *M. flavescens* c27j1^T^ [17] and *M. aerophila* DSM 21344^T^ [10], as well as for the type species of the genus *M. subterranea* DSM 14364^T^ [11], supporting the classification of the strain within the genus *Microvirga*. However, the fatty acid profile of this strain significantly differs from that of the closest related species (Table 4), supporting the classification of CDVBN77^T^ into a different species within the genus.

Phenotypic features analyzed in strain CDVBN77^T^ are detailed in the species protologue, and the main phenotypic differences with the closest related species and the type species of the genus are summarized in Table 5. 

## 4. Discussion

Many plant endophytic strains have been described as new taxa [57,58,59,60]. In this study, we isolated strain CDVBN77^T^, whose 16S rRNA gene sequence showed a relatively low similarity with its closest related species, *M. aerophila* DSM 21344^T^ (97.64%) and *M. flavescens* c27j1^T^ (97.50%), which suggested that it could belong to a new species within the genus *Microvirga*. Several *Microvirga* species, namely, *M. lupini*, *M. lotononidis*, *M. zambiensis*, *M. ossetica*, and *M. vignae* [18,19,20], were for the first time isolated as legume endophytes. The phylogenetic analysis of this strain based on the 16S rRNA gene showed that the bacterium appears in a separated branch within the genus *Microvirga* but clustered with its two closest related type strains *M. flavescens* c27j1^T^ and *M. aerophila* DSM 21344^T^. The presence of Q-10 as the major quinone and C19:0 cyclo ω8c and Summed Feature 8 as the main fatty acids supports its classification within the genus *Microvirga*. However, this isolate differs from the closest species, *M. aerophila* and *M. flavescens*, in the capability to hydrolyze gelatin and aesculin, positive for *M. brassicacearum* CDVBN77^T^ and negative for the other two, and the color of the colonies in R2A agar medium, transparent in *M. brassicacearum* CDVBN77^T^ and light yellow and pale pink in *M. flavescens* and *M. aerophila*, respectively. Unlike *M. aerophila*, it reduces nitrates to nitrites and assimilates D-glucose and L-arabinose, and it does not produce the enzyme β-glucoronidase; moreover, in contrast to *M. flavescens*, it produces trypsin. Thus, the results of the phylogenetic, chemotaxonomic, and phenotypic analysis clearly support the classification of strain CDVBN77^T^ as a new species within the genus *Microvirga*, for which the name *Microvirga brassicacearum* sp. nov. is proposed.

The genus *Microvirga* belongs to Rhizobiales, a broadly studied order in the field of plant–bacteria interactions because of its role as plant growth promoter. Five species of the genus *Microvirga* have been described as plant endosymbionts capable of promoting plant growth, namely, *M. lupini*, *M. lotononidis*, *M. zambiensis* [18], *M. vignae* [20], and *M. ossetica* [19]. The genome mining of strain *M. brassicacearum* CDVBN77^T^ shows the presence of genes implicated in the nutrient provision to the plant, such as P and Fe, capabilities also proven in in vitro tests, as well as K and N. Moreover, both the analysis of the genome sequence as well as in vitro tests show the capability of this strain to synthesize exopolysaccharides which may allow the attachment of the bacterial cells to the plant root surface in order to establish an interaction with the host.

To live as plant endophytes, bacteria must have genetic machinery to use plant-synthesized compounds as a source of nutrients. Some of the enzymes implicated in plant compound degradations are very interesting for industries based on biomass degradation, such as bioethanol production, pulp and paper industries, beverages industries, medicinal and analytical chemistry, fabrication of detergents, and textile de-sizing [6]. Thus, we explored the enzymatic machinery related to carbohydrate degradation encoded by the *M. brassicacearum* CDVBN77^T^ genome. As a result, we identified genes related to the degradation and modification of biomass polymers, such as cellulose and hemicelluloses. β-glucosidase is the key enzyme in the cellulase system [34,61,62], since it converts cellobiose to glucose, completing the final step during cellulose hydrolysis. In the genome of *M. brassicacearum* CDVBN77^T^, we have found five genes belonging to family GH1 and three genes belonging to family GH3; both enzyme families include enzymes, such as β-glucosidases (EC 3.2.1.21) or even exoglucanases (EC 3.2.1.74), enzymes which are also involved in the cellulose polymer degradation [63]. 

The complete degradation of hemicelluloses requires the combined activity of xylanases, β-xylosidases, and several accessory enzymes such as α-arabinofuranosidases [64]. In the genome of *Microvirga brassicacearum* CDVBN77^T^, we have found gene counts in the GH1, GH2, and GH3 families, all of them including enzymes with xylan 1,4-β-xylosidase (EC 3.2.1.37) activity. Moreover, GH2 and G3 families include enzymes with α-arabinofuranosidase activity. However, we could not detect the in vitro synthesis of xylosidases and arabinofuranosidases, either because the annotated genes belonging to the GH1, GH2, and GH3 families have other different activities, or because they were not induced under the assayed conditions.

On the other hand, we found how this bacterium is able to hydrolyze several substrates linked to *p*-nitrophenol, which reveals that the bacterium synthesizes α-rhamnosidase, with potential applications in the clarification and debittering of citrus juices, the enhancement of wine aromas, or the synthesis of certain pharmaceutical compounds [65] and β-galactosidase, with potential application in food processing industries for the hydrolysis of lactose in milk and milk by-products [66].

Regarding the secondary metabolism prospection of the bacterium, we found an interesting niche for researching secondary metabolite BGCs that have not been very studied within the *Microvirga* genus. AntiSMASH results showed that the CDVBN77^T^ strain has the potential to encode at least five undescribed secondary metabolites; amongst those, a terpene, a PKS, and two NRPS have potential applications in industry. NRPS are widely distributed among many taxa, and their products, nonribosomal peptides, are characterized because of their broad range of biological activities, such as being mainly antimicrobials, but also antitumoral compounds, antivirals, siderophores, etc. [67,68,69,70]. The same fact happens with type III polyketides, in which we also found interesting activities [54,71,72]. In addition, carotenoids (terpenes) are also used in the pharmaceutical and food industries because of their antioxidant activities or colorant properties [73,74].

Thus, in this study we found an interesting genetic potential in the genome of *M. brassicacearum* CDVBN77^T^ to synthesize enzymes and secondary metabolites. Moreover, we detected in vitro the production of some of the predicted enzymes and metabolites. Nevertheless, the purification of such gene products and the determination of their specific mechanisms of action in addition to the conditions of pH and temperature in which the molecules are active are required in order to determine the possible industrial interest of those substances.

## 5. Description of *Microvirga brassicacearum* sp. nov.

*Microvirga brassicacearum* (bras.si.ca.ce.a.rum. M.L. fem. pl. gen. n. brassicacearum of the Brassicaceae, referring to its isolation from the rhizoplane of plants belonging to the genus *Brassica*). Cells are rod-shaped, 1.2-1.5 µm in length and 0.7-0.9 µm in width, and motile by means of a polar flagellum. In 869(1/10) medium, colonies are transparent, circular, and flat, with an average size of 0.1–0.4 mm in diameter after seven days of growth at 28 °C in R2A medium. The temperature growth ranges between 12 and 37 °C, with an optimum of 28 °C. The growth at different pH ranges occurs between 6 and 10 with an optimum of 7. It grows between 0 and 1.5% NaCl in 869(1/10) liquid medium. Catalase and oxidase positive. The main polar lipids are phosphatidylcholine and phosphatidylethanolamine. Ubiquinone-10 (Q-10) is the major respiratory quinone and C19:0 cyclo ω8c and Summed Feature 8 are the main fatty acids. Results obtained in the API 20NE system indicate that the strain hydrolyzes aesculin, but it does not ferment D-glucose and does not hydrolyze arginine, urea, and gelatin. Reduction of nitrates to nitrites is positive. The assimilation of D-glucose, L-arabinose, D-mannitol, N-acetyl-glucosamine, D-maltose, potassium gluconate, malic acid, and trisodium citrate is positive and that of D-mannose, capric acid, adipic acid, and phenylacetic acid is negative. Results obtained in the API ZYM system showed activity in the enzymes alkaline phosphatase, esterase (C 4), esterase lipase (C 8), leucine arylamidase, trypsin, acid phosphatase, and naphtol-AS-BI-phosphohydrolase, but activity was not shown in lipase (C14), valine arylamidase, cysteine arylamidase, α-chymotrypsin, α-galactosidase, β-galactosidase, β-glucuronidase, β-glucosidase, α-glucosidase, N-acetyl-β-glucosaminidase, α-mannosidase, and α-fucosidase. 

The G+C base composition was 62.3 mol %. The type strain, CDVBN77^T^ (= LMG 31419^T^= CECT 9905^T^), was isolated as an endophyte from *Brassica napus* roots in Castellanos de Villiquera (Spain).

## Figures and Tables

**Table 1:** Substrates for enzymatic enzymes.

Substrate	Enzyme Assayed
PNP-α-L-arabinopyranoside	α-L-arabinosidase
PNP-β-L-arabinopyranoside	β-L-arabinosidase
PNP-β-D-cellobioside	Cellobiohydrolase
PNP-phosphate (at pH 5.0)	Phosphatase
PNP-phosphate (at pH 7.0)	Phosphatase
PNP-phosphate (at pH 8.5)	Phosphatase
PNP-bis-phosphate (at pH 7)	Bisphosphatase
PNP-bisphosphate (at pH 8.5)	Bisphosphatase
PNP-α-L-fucopyranoside	α-L-fucosidase
PNP-β-D-fucopyranoside	β-glucosidase
PNP-α-D-galactopyranoside	α-galactosidase
PNP-β-D-galactopyranoside	β-galactosidase
PNP-β-D-galacturonide	β-galacturonidase
PNP-α-D-glucopyranoside	α-glucosidase
PNP-β-D-glucopyranoside	β-glucosidase
PNP-β-D-glucuronide	β-glucuronidase
PNP-β-D-lactopyranoside	β-lactosidase
PNP-α-D-mannopyranoside	α-mannosidase
PNP-β-D-mannopyranoside	β-mannosidase
PNP-α-L-rhamnopyranoside	α-rhamnosidase
PNP-N-thio-β-D-glucopyranoside	β-glucosidase
PNP-α-D-xylopyranoside	α-xylosidase
PNP-β-L-xylopyranoside	β-xylosidase

**Table 2:** Genome properties of *Microvirga brassicacearum* CDVBN77^T^.

Attributes	Value
Genome size (bp)	5,221,427
G+C content (%)	62.3
N50 value	130,073
L50 value	12
Number of contigs (with PEGs)	88
Number of subsystems	335
Number of coding sequences	5,244
Number of RNAs	51
Number of genes related to:	
Cofactors, vitamins, prosthetic groups, pigments	151
Cell wall and capsule	45
Virulence, disease, and defense	55
Potassium metabolism	8
Miscellaneous	26
Phages, prophages, transposable elements, plasmids	16
Membrane transport	179
Iron acquisition and metabolism	10
RNA metabolism	41
Nucleosides and nucleotides	96
Protein metabolism	187
Motility and chemotaxis	16
Regulation and cell signaling	47
Secondary metabolism	6
DNA metabolism	94
Fatty acids, lipids, and isoprenoids	109
Nitrogen metabolism	39
Dormancy and sporulation	1
Respiration	109
Stress response	66
Metabolism of aromatic compounds	56
Amino acids and derivatives	367
Sulfur metabolism	42
Phosphorus metabolism	39
Carbohydrates	267

**Table 3:** Predicted gene modules for carbohydrate-active enzymes (CAZymes) families in the genome of *Microvirga brassicacearum* CDVBN77^T.^

CAZyme Family	Gene Count	Known CAZyme Activities
**GHs**		
1	5	β-glucosidase (EC 3.2.1.21); β-galactosidase (EC 3.2.1.23); β-mannosidase (EC 3.2.1.25); β-glucuronidase (EC 3.2.1.31); β-xylosidase (EC 3.2.1.37); β-D-fucosidase (EC 3.2.1.38); exo-β-1,4-glucanase (EC 3.2.1.74); 6-phospho-β-galactosidase (EC 3.2.1.85); and others
2	2	β-galactosidase (EC 3.2.1.23); β-mannosidase (EC 3.2.1.25); β-glucuronidase (EC 3.2.1.31); α-L-arabinofuranosidase (EC 3.2.1.55); exo-β-glucosaminidase (EC 3.2.1.165); α-L-arabinopyranosidase (EC 3.2.1.-); β-galacturonidase (EC 3.2.1.-); β-xylosidase (EC 3.2.1.37)
3	2	β-glucosidase (EC 3.2.1.21); xylan 1,4-β-xylosidase (EC 3.2.1.37); β-glucosylceramidase (EC 3.2.1.45); β-N-acetylhexosaminidase (EC 3.2.1.52); α-L-arabinofuranosidase (EC 3.2.1.55); glucan 1,3-β-glucosidase (EC 3.2.1.58); glucan 1,4-β-glucosidase (EC 3.2.1.74); and others
15	1	glucoamylase (EC 3.2.1.3); glucodextranase (EC 3.2.1.70); α,α-trehalase (EC 3.2.1.28); dextran dextrinase (EC 2.4.1.2)
16	3	xyloglucan:xyloglucosyltransferase (EC 2.4.1.207); keratan-sulfate endo-1,4-β-galactosidase (EC 3.2.1.103); endo-1,3-β-glucanase (EC 3.2.1.39); endo-1,3(4)-β-glucanase (EC 3.2.1.6); licheninase (EC 3.2.1.73); and others
23	3	lysozyme type G (EC 3.2.1.17); peptidoglycan lyase (EC 4.2.2.1), also known in the literature as peptidoglycan lytic transglycosylase; chitinase (EC 3.2.1.14)
25	1	lysozyme (EC 3.2.1.17)
31	3	α-glucosidase (EC 3.2.1.20); α-galactosidase (EC 3.2.1.22); α-mannosidase (EC 3.2.1.24); α-1,3-glucosidase (EC 3.2.1.84); sucrase-isomaltase (EC 3.2.1.48); α-xylosidase (EC 3.2.1.177); α-glucan lyase (EC 4.2.2.13); and others
63	1	α-glucosidase (EC 3.2.1.106); α-1,3-glucosidase (EC 3.2.1.84); α-glucosidase (EC 3.2.1.20); mannosylglycerate α-mannosidase / mannosylglycerate hydrolase (EC 3.2.1.170)
102	1	peptidoglycan lytic transglycosylase (EC 3.2.1.-)
103	3	peptidoglycan lytic transglycosylase (EC 3.2.1.-)
105	1	unsaturated rhamnogalacturonyl hydrolase (EC 3.2.1.172); d-4,5-unsaturated β-glucuronyl hydrolase (EC 3.2.1.-); d-4,5-unsaturated α-galacturonidase (EC 3.2.1.-)
108	1	N-acetylmuramidase (EC 3.2.1.17)
109	2	α-N-acetylgalactosaminidase (EC 3.2.1.49)
113	1	β-mannanase (EC 3.2.1.78)
**PLs**		
9	1	pectate lyase (EC 4.2.2.2); exopolygalacturonate lyase (EC 4.2.2.9); thiopeptidoglycan lyase (EC 4.2.2.-)
20	1	endo-β-1,4-glucuronan lyase (EC 4.2.2.14)
**GTs**		
1	1	UDP-glucuronosyltransferase (EC 2.4.1.17); zeatin O-β-xylosyltransferase (EC 2.4.2.40); 2-hydroxyacylsphingosine 1-β-galactosyltransferase (EC 2.4.1.45); N-acylsphingosine galactosyltransferase (EC 2.4.1.47); and others
2	11	cellulose synthase (EC 2.4.1.12); chitin synthase (EC 2.4.1.16); dolichyl-phosphate β-D-mannosyltransferase (EC 2.4.1.83); dolichyl-phosphate β-glucosyltransferase (EC 2.4.1.117); N-acetylglucosaminyltransferase (EC 2.4.1.-); and others
4	19	sucrose synthase (EC 2.4.1.13); sucrose-phosphate synthase (EC 2.4.1.14); α-glucosyltransferase (EC 2.4.1.52); lipopolysaccharide N-acetylglucosaminyltransferase (EC 2.4.1.56); phosphatidylinositol α-mannosyltransferase (EC 2.4.1.57); and others
19	1	lipid-A-disaccharide synthase (EC 2.4.1.182)
20	1	α,α-trehalose-phosphate synthase [UDP-forming] (EC 2.4.1.15); glucosylglycerol-phosphate synthase (EC 2.4.1.213); trehalose-6-P phosphatase (EC 3.1.3.12); [retaining] GDP-valeniol: validamine 7-phosphate valeniolyltransferase (EC 2.-.-.-)
27	1	polypeptide α-N-acetylgalactosaminyltransferase (EC 2.4.1.41)
28	2	1,2-diacylglycerol 3-β-galactosyltransferase (EC 2.4.1.46); 1,2-diacylglycerol 3-β-glucosyltransferase (EC 2.4.1.157); UDP-GlcNAc: Und-PP-MurAc-pentapeptide β-N-acetylglucosaminyltransferase (EC 2.4.1.227); digalactosyldiacylglycerol synthase (EC 2.4.1.241)
30	1	CMP-β-KDO: α-3-deoxy-D-manno-octulosonic-acid (KDO) transferase (EC 2.4.99.-)
51	6	murein polymerase (EC 2.4.1.129)
83	1	undecaprenyl phosphate-α-L-Ara4N: 4-amino-4-deoxy-β-L-arabinosyltransferase (EC 2.4.2.43); dodecaprenyl phosphate-β-galacturonic acid: lipopolysaccharide core α-galacturonosyl transferase (EC 2.4.1.-)
94	2	GDP-Man: GlcA-β-1,2-Man-α-1,3-Glc-β-1,4-Glc-α-1-PP-undecaprenol β-1,4-mannosyltransferase (EC 2.4.1.251)
**CEs**		
1	4	acetyl xylan esterase (EC 3.1.1.72); cinnamoyl esterase (EC 3.1.1.-); feruloyl esterase (EC 3.1.1.73); carboxylesterase (EC 3.1.1.1); S-formylglutathione hydrolase (EC 3.1.2.12); diacylglycerol O-acyltransferase (EC 2.3.1.20); trehalose 6-O-mycolyltransferase (EC 2.3.1.122)
4	8	acetyl xylan esterase (EC 3.1.1.72); chitin deacetylase (EC 3.5.1.41); chitooligosaccharide deacetylase (EC 3.5.1.-); peptidoglycan GlcNAc deacetylase (EC 3.5.1.-); peptidoglycan N-acetylmuramic acid deacetylase (EC 3.5.1.-)
9	2	N-acetylglucosamine 6-phosphate deacetylase (EC 3.5.1.25); N-acetylglucosamine 6-phosphate deacetylase (EC 3.5.1.80)
10	6	arylesterase (EC 3.1.1.-); carboxyl esterase (EC 3.1.1.3); acetylcholinesterase (EC 3.1.1.7); cholinesterase (EC 3.1.1.8); sterol esterase (EC 3.1.1.13); brefeldin A esterase (EC 3.1.1.-)
11	1	UDP-3-0-acyl N-acetylglucosamine deacetylase (EC 3.5.1.-)
14	1	N-acetyl-1-D-myo-inosityl-2-amino-2-deoxy-α-D-glucopyranoside deacetylase (EC 3.5.1.89); diacetylchitobiose deacetylase (EC 3.5.1.-); mycothiol S-conjugate amidase (EC 3.5.1.-)
**AAs**		
3	5	cellobiose dehydrogenase (EC 1.1.99.18); glucose 1-oxidase (EC 1.1.3.4); aryl alcohol oxidase (EC 1.1.3.7); alcohol oxidase (EC 1.1.3.13); pyranose oxidase (EC 1.1.3.10)
4	6	vanillyl-alcohol oxidase (EC 1.1.3.38)
6	1	1,4-benzoquinone reductase (EC 1.6.5.6)
7	2	glucooligosaccharide oxidase (EC 1.1.3.-); chitooligosaccharide oxidase (EC 1.1.3.-)
12	1	pyrroloquinoline quinone-dependent oxidoreductase

**Table 4:** Cellular fatty acid composition (%) of *Microvirga brassicacearum* CDVBN77^T^ (data from this study) and its closest related species *M. flavescens* c27j1^T^ (data from Zhang et al. [17]) and *M. aerophila* DSM 21344^T^ (data from Weon et al. [10]), as well as for the type species of the genus *M. subterranea* DSM 14364^T^ (data from Kanso and Patel [11]). Strains: 1, *M. brassicacearum* CDVBN77^T^; 2, *M. flavescens* c27j1^T^; 3, *M. aerophila* DSM 21344^T^; 4, *M. subterranea* DSM 14364^T^.

Fatty Acid	1	2	3	4
C_16:0_	9.2	4.8	6.6	7.6
C_17:0_			tr	7.5
C_18:0_	6.1	2.0	3.1	5.5
C_17:0_ cyclo	3.8	3.5	2.1	tr
_19:0_ cyclo ω8c	24.3	57.7	11.8	27.9
C_20:2_ ω6,9c		1.1	tr	1.1
11-Methyl C_18:1_ ω7c	4.2	1.5	tr	1.5
C_18:0_ 3-OH	1.7	2.1	1.1	1.7
Summed feature 2	4.5	5.0	1.4	3.2
Summed feature 3	4.5	1.1	5.2	1.1
Summed feature 8	39.3	18.5	64.8	38.5

The values are percentages of the total fatty acids. The values under 1% for all of the strains are not included. tr: traces, values under 1%. Summed feature 2: C12:0 aldehyde and/or unknown 10.9525. Summed feature 3: C_16:1_ ω7c and/or C_16:1_ ω6c. Summed feature 8: C_18:1_ ω7c and/or C_18:1_ ω6c.

**Table 5:** Phenotypic differences between *Microvirga brassicacearum* CDVBN77^T^ (data from this study) and its closest related species *M. flavescens* c27j1^T^ (data from Zhang et al. [17]) and *M. aerophila* DSM 21344^T^ (data from Weon et al. [10]), as well as for the type species of the genus *M. subterranea* DSM 14364T (data from Kanso and Patel [11]) Strains: 1, *M. brassicacearum* CDVBN77^T^; 2, *M. flavescens* c27j1^T^; 3, *M. aerophila* DSM 21344^T^; 4, *M. subterranea* DSM 14364^T^. +, positive; w, weakly positive; -, negative.

Characteristic	1	2	3	4
Isolation source	Plant	Soil	Air	Thermal aquifer
Colony color	White	Faint yellow	Light pink	Light pink
Motility	+	+	−	−
Oxidase	+	+	+	−
Nitrate reduction	+	+	−	+
Hydrolysis of:				
Gelatin	+	−	−	+
Aesculin	+	−	−	−
Assimilation of:				
D-Glucose	+	+	−	−
L-Arabinose	+	+	−	+
Production of:				
β-glucoronidase	−	−	+	−
Esterase lipase (c8)	+	w	+	+
Trypsin	+	−	+	w
pH range	6–10	6–10	7–10	6–9
Salinity range (%)	0–1.5	0–1	0–2	0–1
Temperature range (°C)	12–37	15–37	10–35	25–45
DNA G+C content (mol %)	62.3	62.2	62.1	65.1
